# Time Savings Through an AI Speech Assistant for Nursing Documentation: Pre-Post Time-Motion Study in German Long-Term Care

**DOI:** 10.2196/86078

**Published:** 2026-04-08

**Authors:** Katja Schwabe, Drin Ferizaj, Susann Neumann, Sandra Strube-Lahmann, Nils Lahmann

**Affiliations:** 1 voize GmbH, Dircksenstraße 47, Berlin, 10178, Germany, +49 331 96809186; 2 Department of Geriatrics and Medical Gerontology, Charité – Universitätsmedizin Berlin, Berlin, Germany

**Keywords:** nursing documentation time, automatic speech recognition, long-term care, artificial intelligence, AI speech assistant, mobile nursing documentation

## Abstract

**Background:**

Nurses in long-term care spend up to one-third of their working time on documentation, contributing to administrative burden and limited time for direct care. Artificial intelligence (AI) speech assistants have shown potential to accelerate documentation, but longitudinal evidence from real-world long-term care settings remains scarce.

**Objective:**

This study aimed to evaluate whether implementing a domain-specific, mobile AI speech assistant is associated with reduced documentation time in German long-term care under routine conditions. Secondary objectives included examining usability, perceived documentation effort, interruptions, and workplace satisfaction.

**Methods:**

A pre-post time-motion study with full-shift observation was conducted. Continuous, event-based observations were performed before (*t*_0_) and after (*t*_1_) implementation of the mobile speech assistant voize. The primary outcome was total documentation time per morning shift based on direct observations. In addition to observations, questionnaires were administered to assess perceived documentation effort, interruptions, satisfaction with the documentation system, and workplace satisfaction. The primary end point was analyzed using a linear mixed-effects model. Secondary, self-reported outcomes were analyzed exploratorily via paired pre-post differences with pooling across multiple imputations and Holm-Bonferroni correction.

**Results:**

A total of 52 registered nurses from 14 long-term care facilities participated (mean age 42.37, SD 12.37 years; 42/52, 80.8% female). Across 770 observed hours, the observed total documentation time per morning shift decreased significantly by an adjusted mean of 15 (SE 3.36) minutes, *t*_46.29_=−4.46, *P*<.001, with a 95% CI of −21.75 to −8.23, corresponding to an approximately 28% reduction relative to the baseline mean. Holm-Bonferroni–corrected exploratory analyses indicated significant declines in self-reported documentation time and interruptions, and satisfaction with the documentation system improved, while workplace satisfaction showed no significant change. Usability was rated as acceptable.

**Conclusions:**

This study provides real-world evidence from a single-group pre-post design that an AI-based speech assistant is associated with reduced documentation workload in long-term care. In this sample, the integration of a mobile, domain-specific speech system into daily workflows coincided with substantially decreased documentation time and improved perceived efficiency. Beyond these observed time savings, such technology has the potential to alleviate workload, free time for resident care, and enhance working conditions. These findings are also relevant for policy discussions on addressing the nursing workforce shortage, showing that well-integrated, speech-enabled documentation systems can support more sustainable long-term care environments.

## Introduction

### Background

In Germany, approximately 1.7 million nursing staff are employed, with nearly 30% working in long-term care facilities, making this the largest sector in the nursing workforce [1]. Demand for care continues to rise while shortages of qualified staff worsen [2]. Nursing staff, particularly in long-term care, report higher levels of strain compared to other occupational groups [3,4]. They work under considerable time pressure, often lack sufficient time to complete tasks, and frequently perform overtime with irregular or missed breaks [3,5]. Alongside these structural challenges and workload demands, care workers face a high administrative burden: documentation tasks account for about 30% of total working time [6,7], leaving less room for direct interaction and care for residents [8]. Furthermore, the high documentation workload has been linked to reduced job satisfaction and an elevated level of stress [9]. In light of these issues, artificial intelligence (AI)–based speech assistants are presented as promising solutions to accelerate the documentation process and improve its accuracy [10].

### Medical Documentation With Speech Recognition

Research on speech recognition has been most widely conducted in clinical medicine, often involving physicians and mixed professional groups. Previous findings show that speech recognition technologies can significantly streamline documentation workflows compared to traditional methods. For example, Zuchowski and Göller [11] reported notable time savings and lower error rates when using speech recognition versus manual typing. Similarly, Peine et al [12] found that a voice-based system enabled faster and more accurate documentation among intensive care professionals compared to both electronic and paper-based approaches.

These findings are further supported by systematic reviews. Alboksmaty et al [13] synthesized findings across several countries, highlighting consistent efficiency gains, reduced administrative burden, and improved provider-patient interaction. Of particular note, Wang et al [14] found that a digital scribe could accelerate history-taking and physical examination documentation by more than double compared to typing or traditional dictation.

However, reviews also point out that most studies have taken place in controlled or experimental settings, which may limit generalizability to routine care [10,13]. In this context, Ng et al [15] reviewed 29 research articles and observed that many reported time savings and improvements in documentation completeness, whereas others described challenges such as increased editing workload, variable cost-effectiveness, and issues with error rates for specialized terminology or accented speech.

### Clinical Nursing Documentation With Speech Recognition

Compared to physician documentation, evidence on nursing documentation with AI speech assistants is more limited but growing. Findings to date are still heterogeneous. For example, many studies reviewed by Joseph et al [10] indicated beneficial effects, such as reduced documentation time and improved workflow. However, a substantial number reported no significant impact, given ongoing challenges integrating AI speech assistants into real-world nursing environments and existing documentation systems.

However, several recent investigations suggest a promising potential for efficiency gains in real-world nursing environments. In a hospital time-motion study, Ehrler et al [16] found that a mobile bedside application meaningfully decreased the duration of electronic health record (EHR) documentation for nurses while also increasing time available for direct patient interaction. Similarly, Mayer et al [17] reported statistically significant time savings—up to 6.1 minutes per documentation scenario—when using a voice recognition system compared to traditional keyboard entry. Mairittha et al [18] also demonstrated that a smartphone-based dialogue system allowed for 15 percent faster documentation relative to electronic forms in a controlled setting. Likewise, qualitative findings from German long-term care facilities hint at the potential of AI speech assistants for care documentation, reporting self-reported time savings, fewer interruptions, greater flexibility, and less reliance on stationary computers [19].

### Research Gap

In summary, evidence across clinical settings suggests that AI speech assistants can shorten documentation time and thus reduce administrative burden. At the same time, findings are mixed and often derive from controlled or pilot evaluations rather than routine practice, which limits generalizability [10,13,15]. In nursing, several studies report faster entries with mobile or dialogue-based tools, but most were scenario-based, single-unit, or acute-care focused, with heterogeneous outcomes and little full-shift observation. Long-term care therefore remains underrepresented despite the high documentation burden [12,16-18].

### Objectives

Therefore, this study aims to address these gaps by evaluating a domain-specific mobile AI speech assistant under routine conditions in German long-term care using full-shift time-motion observation at 2 time points, before and after implementation. The primary end point is the adjusted change in total documentation time per observed morning shift, defined as the cumulative time spent on a set of predefined documentation-related tasks. Secondary outcomes are self-reported and include usability, perceived documentation effort and interruptions, workplace satisfaction, and self-reported documentation quality.

### Hypotheses

The following hypothesis will be tested for the primary end point:

H0: There will be no significant reduction measured by the adjusted mean change in total documentation time from baseline to postimplementation.
H1: There will be a significant reduction measured by the adjusted mean change in total documentation time from baseline to postimplementation.

The secondary outcomes are evaluated exploratorily by pre-post changes. To adjust for multiple testing, the Holm-Bonferroni correction is applied across all secondary tests.

## Methods

### Study Design and Setting

The study was conducted as part of the “PYSA – Nursing Documentation With Hybrid Speech Assistant” project, funded by the German Federal Ministry of Education and Research [20]. The longitudinal research design follows a pre-post observational time-motion design with 2 measurement points (baseline *t*_0_ and postassessment *t*_1_) and takes place in stationary long-term care facilities in Germany. Facilities were approached nationwide to ensure the representation of different conditions and to include both rural and urban areas as well as different types of providers. All participating facilities used either the same EHR system or paper-based documentation.

The minimum age for inclusion of participants was 18 years, with no upper age limit. Additional inclusion criteria were a completed 3-year nursing education, regular involvement in nursing documentation, and at least 1 year of professional experience. Participants had to be able to understand instructions in German and provide informed consent. Only staff employed in long-term care facilities in which the speech assistant voize was implemented during the study period were eligible. Exclusion criteria were applied to staff who were not permanently employed in the participating facilities, such as agency or temporary nurses. We report this observational study in accordance with the STROBE (Strengthening the Reporting of Observational Studies in Epidemiology) statement [21].

### Recruitment

Participants were recruited through long-term care facilities that planned to implement the speech assistant voize and received written information about the study procedures. Following approval from the employee representative bodies of each facility, nursing staff were approached by the facility management. All participants were informed in writing that participation was voluntary, that data would be pseudonymized and analyzed in aggregated form, and that they could withdraw from the study at any time without facing any negative consequences. Participants were offered a voluntary small financial compensation for their time and effort.

### The Speech Assistant Voize

The speech assistant voize is a software-based mobile app that enables users to dictate nursing documentation via smartphone. It uses a continuously trained language model that processes voice inputs locally, assigns the content to the appropriate categories, and automatically generates structured documentation entries. These entries are reviewed and approved by nursing staff before being integrated into the existing documentation system. Developed in close collaboration with nursing professionals [19,20], the application is continuously refined through a collaborative, user-centered design process, and its underlying models are regularly trained with voluntarily submitted and anonymized documentation data to ensure ongoing improvements and adaptation to the linguistic characteristics and professional terminology used in nursing.

Technically, the system converts spoken language into text (speech-to-text) in real time using an end-to-end streaming ASR (automatic speech recognition) model. In conjunction with this, a set of specialized natural language processing models is activated as needed to handle specific tasks—such as understanding context, matching spoken language to planned interventions, extracting key information, or structuring data—ensuring that all components work together to deliver the supported features. As part of the study, voize was introduced in cooperation with voize GmbH. The implementation included planning and rollout within the facilities.

### Power Analysis

In a preliminary anonymous survey with 55 nurses already using voize, about 70% reported subjective time savings of 16 to 30 minutes per shift. It was assumed that about one-third of working time is spent on documentation-related tasks [6,7], which is equivalent to about 20 minutes per hour. A 15% reduction was considered meaningful, corresponding to the mean of reported savings (23 minutes per shift), that is, 2.9 minutes less per working hour, reducing documentation from 20 to 17.1 minutes per hour. With an assumed SD of 6 minutes, this equates to an effect size of Cohen *d*=0.5. Power analysis using the R package pwr [22] (α=.05, 2-sided; power=0.8) indicated a minimum of 34 participants for paired *t* tests. To account for an expected 15% dropout, the target sample size was set at 40, compensating for likely reasons such as scheduling conflicts, shift changes, sick leave, or vacation.

Early-stage dropout rates exceeded expectations. Therefore, additional recruitment efforts were employed. Nonetheless, interest from both facilities and individual nurses was high, and incremental participant burden remained low. As a result, a total of 52 nurses were included in this study.

### Measures

#### Primary End Point: Total Documentation Time per Shift

The primary outcome was total documentation time per observed morning shift in minutes, defined as the sum of all documentation-related categories recorded during the time-motion observation. Observation categories were developed and refined during preparatory nonparticipant observations in a long-term care facility within an unpublished pilot study [20]. The identified documentation-related tasks included documentation at the PC or on paper, consulting information at the resident record at the PC, waiting for a PC, booting up and logging into the PC, walking to the ward office for documentation purposes, making notes, and reviewing entries (Table S1 in Multimedia Appendix 1). All facilities applied their usual infection control protocols for mobile devices. Nurses were instructed to disinfect the study smartphones in accordance with local hygiene guidelines. When these hygiene steps occurred in direct connection with documentation, they were included in the observed documentation time. At *t*_1_, after implementation of the application, additional categories were included for documentation with voize and consulting information in voize (Table S2 in Multimedia Appendix 1).

#### Nursing Workplace Satisfaction Questionnaire (NWSQ)

Workplace satisfaction outcomes were assessed using the NWSQ [23]. The NWSQ is a brief measure of nursing job satisfaction encompassing 3 domains: intrinsic, extrinsic, and relational satisfaction. Items are rated on a 5-point Likert scale, and domain scores are computed as the sum of their constituent items. Possible ranges are 6‐30 for intrinsic, 5‐25 for extrinsic, and 4‐20 for relational satisfaction, with higher scores indicating greater satisfaction.

#### Usability

Technology-related usability was measured with the system usability scale [24] (SUS), a 10-item questionnaire of perceived usability on a 5-point Likert scale and alternating item valence. Responses were scored using the standard SUS procedure, including appropriate reverse-scoring, and converted to a total score from 0 to 100. Scores around 70 indicate acceptable usability, with higher values reflecting better perceived usability [24].

#### Self-Developed Questionnaire

Several single-item measures were developed and used to capture several facets of potential impact based on a previous study [19]. Items included perceived interruptions during daily work, the perceived time-consuming nature of documentation, satisfaction with the documentation system, expected quality improvement, expected time savings, current documentation quality and completeness, implementation as a good idea, intention-to-use, and self-reported documentation time in minutes per shift. Items used 5-point scales or 10-point scales as shown in the Results section, plus open numerical entries for minutes. Higher ratings indicate more agreement with the item. For example, higher values on “ documentation is time-consuming” indicate greater perceived time burden, and higher values on “satisfaction with the documentation system” indicate greater satisfaction. Item wording and differences across *t*_0_ and *t*_1_ are provided in Tables S1 and S2 in Multimedia Appendix 2.

### Procedure

Data were collected between September 2024 and July 2025 in German long-term care facilities. Trained observers conducted continuous, nonparticipant full-shift observations of morning shifts with a planned duration of 8 hours, and observed shift lengths were very similar between *t*_0_ and *t*_1_.

Time was captured with an event-based smartphone logging app by starting and stopping predefined documentation-related task categories. After completing the shift, participants were asked to complete an online questionnaire that included the measures.

Approximately 8 weeks after the implementation of voize (*t*_1_), participants were observed again during a full morning shift, and documentation times were recorded using the same procedure. At this second measurement point, participants completed an adjusted online survey, including the SUS.

Observations at both time points took place on typical weekdays outside major holiday periods. According to the participating units, bed capacity and staffing ratios were generally stable between *t*_0_ and *t*_1_. Taken together, these conditions suggest that major systematic differences in resident census between *t*_0_ and *t*_1_ were unlikely, although smaller variations may still have occurred.

### Observers and Minimization of Bias

Prior to data collection, all observers were employees of voize GmbH who were specifically hired for the purpose of this study and were not involved in the development or design of the speech assistant. Observers had no performance incentives tied to study outcomes and were instructed to adhere strictly to the predefined task categories and timing rules to minimize the risk of biased classification or timing cessation. They received standardized training from the Charité research team in the use of the measurement instruments and the predefined task categories. As part of this training, observers first jointly worked through standardized observation scenarios and coded them in parallel. Any discrepancies were then discussed systematically until a shared consensus on the application of each category was reached. In addition, weekly meetings were held throughout the data collection period to address questions, reflect on measurement procedures, and resolve ambiguities in a standardized manner. During the observations, observers also had access to a notes field where they could record questions or uncertainties regarding the assignment to task categories, add comments, and briefly describe the situation. This enabled the study personnel to later compare the selected task category with the descriptive notes and to correct obvious misclassifications where necessary.

In addition, the direct observation design can lead to behavioral changes among participants. The presence of an external observer can sometimes prompt improved performance, a phenomenon known as the Hawthorne effect [25]. To mitigate such influences, repeated briefings and transparent communication of the study objectives were provided to the nursing staff and critical questions were encouraged.

### Statistical Analysis

Statistical analyses were performed using Python 3.11.9 and R 4.5.2 in Visual Studio Code 1.103.2 with the packages scikit-learn [26], miceforest [27], NumPy [28], pandas [29], SciPy [30], Matplotlib [31], tidyverse [32], lme4 [33], and lmerTest [34]. All statistical methods used 2-sided tests at α=.05.

To account for the hierarchical and longitudinal structure of the data with repeated measurements within participants and clustering within facilities, the primary end point was analyzed using a linear mixed-effects model (LMM). The primary estimand was the adjusted mean change in documentation time from baseline to postimplementation (*t*_1_ – *t*_0_) in minutes. Time was coded as 0 at baseline and 1 at postimplementation, so that the fixed effect of time directly represents the adjusted mean change. The primary model included fixed effects for time, age, gender, and baseline documentation type, and random intercepts for participants and facilities. The model was estimated with restricted maximum likelihood. Inference for fixed effects used Kenward-Roger degrees of freedom.

The primary model used all available outcome observations and handled missing primary outcomes via likelihood-based estimation under a missing-at-random assumption. Robustness was assessed in exploratory sensitivity analyses, including a complete-case analysis requiring observations at both time points, models additionally accounting for observer effects, and alternative random-effects specifications. We further repeated analyses using multiple imputation with pooling via Rubin rules and Barnard-Rubin degrees of freedom and conducted missing-not-at-random (MNAR) pattern-mixture sensitivity analyses including jump-to-baseline and delta adjustments. As an additional robustness check, we repeated the analysis using a baseline-adjusted analysis of covariance with change scores. Full model specifications, statistical rationale, imputation settings, and MNAR sensitivity procedures are provided in Multimedia Appendix 3. As the primary and secondary analyses fall under the frequentist paradigm, we additionally conducted an exploratory Bayesian hierarchical repeated-measures analysis (Multimedia Appendix 4).

All secondary, self-reported outcomes were analyzed exploratorily using within-subject pre-post comparisons on multiply imputed datasets pooled according to Rubin rules and Barnard-Rubin small sample adjustment. For each outcome, the estimand was the mean change score (*t*_1_ – *t*_0_) with heteroscedasticity-consistent type 3 robust standard errors. To account for multiplicity, a Holm-Bonferroni correction was applied across secondary outcomes.

### Ethical Considerations

The study was reviewed and approved by the Ethics Committee of Charité – Universitätsmedizin Berlin, approval number EA1/209/24. All participants provided written informed consent prior to participation. Data were pseudonymized and analyzed in aggregated form. The study was preregistered in the German Clinical Trials Register (DRKS00035512). Participants received 30 € (US $34.43) for their participation.

## Results

### Participant Characteristics

The sample comprised 52 registered nurses from 14 long-term care facilities in Germany. Of the 52 participants, 8 (15.4%) dropped out between baseline and postassessment, primarily due to long-term illness or resignation.

The mean age was 42.37 (SD 12.37) years, with 17.52 (SD 9.63) years of professional experience and 9.42 (SD 8.13) years of tenure. Most participants were female (42/52, 80.8%). Among completers (44/52, 84.6%), the mean age was 43.23 (SD 12.01) years and mean experience was 18.2 (SD 9.95) years; 84.1% (37/44) were female. Dropouts (8/52, 15.4%) were younger on average (mean age 37.62, SD 14.07 years) and reported less work experience (mean 13.75, SD 6.88 years), and 5/8 (62.5%) of them were female. Detailed counts and percentages for all characteristics are provided in Table 1.

**Table 1.** Participant characteristics of the total sample, completers, and dropouts.

Variable	Total (N=52)	Completers (n=44)	Dropouts (n=8)
Age (years), mean (SD)	42.37 (12.37)	43.23 (12.01)	37.62 (14.07)
Work experience (years), mean (SD)	17.52 (9.63)	18.20 (9.95)	13.75 (6.88)
Tenure (years), mean (SD)	9.42 (8.13)	9.75 (8.41)	7.62 (6.52)
Gender, n (%)			
Male	10 (19.2)	7 (15.9)	3 (37.5)
Female	42 (80.8)	37 (84.1)	5 (62.5)
Other	0 (0)	0 (0)	0 (0)
Role, n (%)			
Registered nurse	52 (100)	44 (100)	8 (100)
Leadership role	6 (11.5)	5 (11.4)	1 (12.5)
Practical instructor	6 (11.5)	6 (13.6)	0 (0)
Other	1 (1.9)	0 (0)	1 (12.5)

### Primary Outcome

#### Descriptive Analyses

At *t*_0_ (n=52), documentation was predominantly PC-based (35/52, 67.3%), followed by hybrid use (11/52, 21.2%) and paper (6/52, 11.5%). Among completers at *t*_1_ (n=44), the speech assistant voize was reported as the primary documentation mode by 18/44 (40.9%), PC for 25/44 (56.8%), and paper by 1/44 (2.3%). Usage frequency of the speech assistant was high: 33/44 (75%) reported daily use, 9/44 (20.5%) used the speech assistant 3‐4 times per week, and 2/44 (4.5%) 1‐2 times per week.

**Figure 1.**
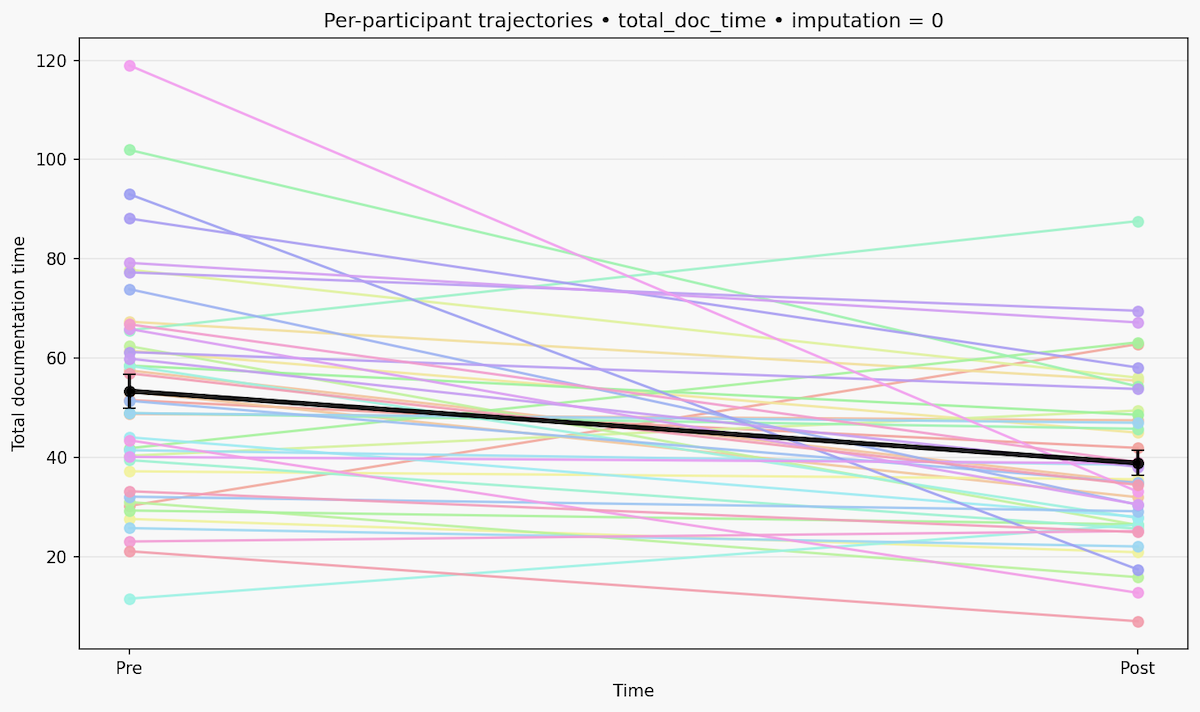
Baseline to postassessment changes in total documentation time among participants.

Overall, around 770 hours of work were observed in long-term care during this study. Total documentation-related time decreased from a mean of 53.91 (SD 23.59) minutes at baseline to 39.10 (SD 16.29) minutes postimplementation in the analysis pooled across 40 multiply imputed datasets (Δ=−14.81 minutes). For the change in total documentation time (post – pre in minutes), complete cases (n=44; see Figure 1) had Q1 –22.48 and Q3 –2.90 (min=−85.90, max=32.60), and the pooled analysis (n=52) had Q1 –23.59 and Q3 –2.54 (min=−86.95, max=32.91).

Descriptive reductions were concentrated in active documentation (Δ=−13.28) and note-taking (Δ=−2.38), while reviewing entries (Δ=+0.64) and information retrieval (Δ=+1.28) showed small descriptive increases, consistent with a shift from producing text to checking/retrieving information.

#### Primary Outcome: Changes in Total Documentation Time

The LMM with random intercepts for participants and facilities and fixed effects for time, age, gender, and baseline documentation type indicated a statistically significant reduction in total documentation time. The adjusted mean change for time (*β*) was −15 (SE 3.36) minutes, *t*_46.29_=−4.46, *P*<.001, with a 95% CI −21.75 to −8.23. Relative to the observed baseline mean of 53.91 minutes, this corresponds to an adjusted 28% reduction in documentation time per morning shift. Variance partitioning indicated an intraclass correlation coefficient of 0.36 at the participant level and 0.08 at the facility level.

Exploratory analyses including time-by-covariate interactions did not indicate reliable heterogeneity of the time effect by age (*P=*.25), gender (*P=*.87), or baseline documentation type (*P=*.49). The estimated reduction was stable across sensitivity analyses, with effect estimates and confidence intervals closely aligned with the primary model. The reduction also remained highly robust under extreme simulated MNAR departures. Detailed results of the exploratory and sensitivity analyses are provided in Multimedia Appendix 5.

### Secondary, Self-Reported Outcomes

There was a significant reduction in self-reported documentation time (Δ=−31.14, SE 6.57 minutes), *t*_43.18_=−4.88, *P*<.001, 95% CI −45.31 to −18.83. Self-reported daily work interruptions also declined, *t*_47.00_=−3.95, *P*<.001, 95% CI −0.95 to −0.31. Participants experienced the documentation process as markedly less time-consuming, *t*_46.91_=−9.32, *P*<.001, 95% CI −1.97 to −1.27, and satisfaction with the documentation system increased after the implementation of voize, *t*_46.47_=4.84, *P*<.001, 95% CI 1.04‐2.52. Expectations that the voice assistant would improve documentation quality were also lower after use, *t*_46.82_=−3.40, *P*=.001, 95% CI −0.75 to −0.19. All 5 of these effects remained significant after Holm-Bonferroni correction across the 13-test family (Holm-adjusted *P*≤.013 for each; see Table 2).

**Table 2.** Pooled measures of central tendency for secondary, self-reported outcomes^a^.

Variables (score range: min-max)	Pooled baseline, mean (SD)	Pooled post, mean (SD)	Mean difference (post – pre)	Median (pre)	Median (post)
Interruptions in daily work (range: 1‐5)	4.06 (0.78)	3.40 (0.91)	−0.66^b^***	4.00	3.97
Self-reported documentation time (in minutes)	67.05 (40.98)	35.91 (24.34)	−31.14^b^***	60.00	30.00
Documentation is time-consuming (range: 1‐5)	4.07 (0.79)	2.46 (0.84)	−1.61^b^***	4.00	2.18
Satisfaction with documentation system (range: 1‐10)	5.07 (2.07)	6.65 (1.82)	+1.58^b^***	5.00	7.00
Expected quality improvement (range: 1‐5)	3.69 (0.74)	3.18 (0.81)	−0.51^b^**	4.00	3.00
Documentation is complete (range: 1‐5)	3.27 (0.85)	2.83 (0.94)	−0.44**	3.00	2.87
Expected time savings (range: 1‐5)	3.81 (0.69)	3.46 (0.79)	−0.35*	4.00	3.57
High documentation quality (range: 1‐5)	3.38 (0.82)	3.03 (0.75)	−0.35*	3.89	3.00
Implementation as a good idea (range: 1‐10)	7.91 (1.93)	7.35 (2.04)	−0.56	8.00	7.73
Intention to use (range: 1‐10)	8.17 (1.99)	7.88 (1.69)	−0.29	8.13	8.00
NWSQ^c^: extrinsic (range: 5‐25)	17.60 (2.34)	17.95 (2.94)	+0.35	17.51	18.00
NWSQ: intrinsic (range: 6‐30)	22.21 (3.94)	22.04 (4.42)	−0.17	22.00	21.63
NWSQ: relational (range: 4‐20)	15.62 (2.62)	15.31 (2.66)	−0.31	16.00	16.00

a All values are pooled across 40 multiply imputed datasets according to Rubin rules. Consequently, some median scores are not restricted to the discrete response categories. The significance of the change is indicated by asterisks based on paired *t* tests. *P<.05; **P<.01; ***P<.001.

b Effects that remained significant after Holm-Bonferroni correction for 13 tests.

c NWSQ: Nursing Workplace Satisfaction Questionnaire.

Perceiving the implementation of voize as a good idea showed no reliable change, *t*_46.29_=−1.09, *P=*.28, 95% CI −1.04 to 0.31, and intention to use remained essentially unchanged at a high level, *t*_46.60_=−0.45, *P*=.65, 95% CI −0.77 to 0.49. Both remain nonsignificant after correction (Holm-adjusted *P*≥.99). Ratings that the current documentation is qualitatively high decreased, *t*_46.97_=−2.30, *P*=.03, 95% CI −0.61 to −0.04, as did ratings that it is complete and comprehensive, *t*_47.01_=–2.75, *P=*.008, 95% CI −0.74 to –0.11, and expected time savings with the speech assistant, *t*_46.74_=–2.60, *P*=.01, 95% CI −0.55 to −0.07. However, none of these 3 effects survived Holm-Bonferroni correction (Holm-adjusted *P=*.16, .06, and .09, respectively).

Nursing workplace satisfaction measured by the NWSQ did not change significantly (extrinsic: *t*_38.94_=1.48, *P*=.15, 95% CI −0.22 to 1.45; intrinsic: *t*_39.06_=0.07, *P=*.95, 95% CI −1.03 to 1.10; relational: *t*_38.46_=−0.68, *P*=.5, 95% CI −0.84 to 0.42), and these remain nonsignificant after correction (Holm-adjusted *P*=.73, ≥.99, and ≥.99, respectively). The SUS scores indicated acceptable usability [24]: the complete-case mean was 70.42 (SD 14.26; n=40).

## Discussion

### Principal Findings

This study provides real-world evidence from long-term care that a mobile speech assistant is associated with reduced documentation time over full shifts. The primary LMM estimated an adjusted reduction of approximately 15 minutes per morning shift, corresponding to an approximately 28% adjusted decrease relative to the baseline mean. This finding was robust across extensive sensitivity analyses. The interquartile range indicated that most participants experienced time savings. Task-level patterns suggest that time was recaptured by making active documentation more efficient (see Table 3). However, net time savings may be overestimated as infection control steps such as disinfection time and adherence to infection control protocols were not systematically recorded in this study. In parallel, nurses reported fewer interruptions, lower perceived time burden, and higher satisfaction with the mobile AI speech assistant. Notably, self-reported retrospective time savings were substantially larger than the observed reduction, and perceived baseline documentation time exceeded our time-motion estimates. This divergence suggests that perceived relief in documentation burden may exceed objective time savings when moving from manual entry to speech-based workflows. Retrospective estimates may apply broader boundaries to what counts as documentation and rely on memory-based summary impressions that overweight salient moments. In this context, cognitively exhaustive, interruption-prone work such as administrative documentation may be remembered as more time-consuming, whereas patient interaction may feel more absorbing and therefore subjectively shorter. By contrast, time-motion observation captures only predefined, observable tasks within the sampled shift, yielding an objective estimate of recorded activities that does not necessarily map onto perceived workload.

**Table 3.** Documentation time by task in minutes at baseline and postimplementation.

Task	Pre^a^ (minutes), mean (SD)	Post-complete^b^ (minutes), mean (SD)	Post-pooled^c^ (minutes), mean (SD)
Computer login	1.45 (1.32)	1.05 (1.13)	1.01 (1.09)
Active documentation	44.23 (19.72)	30.44 (14.71)	30.95 (14.36)
Reviewing entries	0.82 (3.65)	1.47 (3.81)	1.46 (3.68)
Information retrieval	3.48 (4.71)	4.85 (6.69)	4.76 (6.48)
Walking to the PC (for documentation)^d^	0.56 (1.56)	0.10 (0.24)	0.09 (0.23)
Taking notes	3.15 (2.68)	0.84 (1.67)	0.77 (1.56)
Waiting	0.22 (0.88)	0.07 (0.33)	0.06 (0.31)
Total documentation-related time	53.91 (23.59)	38.82 (16.75)	39.10 (16.29)

a Pre: observed baseline for all participants (N=52).

b Post-complete: observed postimplementation among completers (n=44).

c Post-pooled: estimates pooled across 40 multiply imputed datasets under “missing at random” assumption (N=52).

d “Walking to the PC (for documentation)” refers exclusively to walking carried out to reach stationary ward computers or the ward office for documentation purposes. Walking that occurred while documentation was being performed (eg, dictating with the mobile speech assistant while moving) was not logged as a separate walking task but was counted only as documentation time.

Overall usability was rated as acceptable. Taken together, these findings suggest that introducing speech-enabled, mobile documentation can reduce documentation time while improving perceived documentation efficiency.

At the same time, self-reported documentation quality and completeness showed a consistent negative trend after implementation. In the frequentist exploratory analyses, these decreases were statistically significant before multiplicity correction, but not after Holm-Bonferroni adjustment. However, the complementary Bayesian analysis used adaptive regularization for secondary outcomes and assessed practical relevance using a region of practical equivalence. The Bayesian analysis still assigned a high posterior probability of negative changes in perceived documentation completeness and quality, while the 94% highest density intervals slightly overlapped zero (see Multimedia Appendix 4). Together, these findings suggest a potential trade-off in routine use. Faster, more mobile documentation may coincide with lower perceived completeness or quality, possibly because of recognition errors, editing burden, or shorter entries [16,18,19].

In stationary long-term care, documentation consumes substantial nurse time. A domain-specific mobile speech assistant may reclaim minutes each shift, possibly reduce interruptions, and improve the overall documentation experience. In labor-constrained markets, recovered time can be redeployed to resident care, coordination, supervision, taking necessary breaks, and improving working conditions while lowering overtime [19].

### Relation to Prior Work

These findings are consistent with experimental and simulation studies showing that speech-based input improves speed and, in some contexts, accuracy. In nursing, Mayer et al [17] found time savings of 2.3 to 6.1 minutes per scenario without increased errors and high user preference. Similarly, Mairittha et al [18] reported 15% faster entries with a dialogue-based system. In addition, another mobile bedside app reduced EHR charting time and increased uninterrupted patient interaction in a pre-post study [16]. In broader clinical samples, speech systems shortened task completion time and reduced error rates versus paper and traditional patient data management systems in intensive care units [12]. In hospital-wide deployments, they also achieved high adoption with large transcription-cost reductions and perceived gains in quality and efficiency [35]. Observational comparisons have also shown that speech can be substantially faster than typing, even when perceptions may differ [11].

Relative to this body of evidence, this study adds three elements. First, it focuses on long-term care, where evidence has been sparse and documentation burden is high. Second, it evaluates the technology under routine conditions, not only in controlled or simulated scenarios. Third, it uses full-shift observation at both time points, yielding approximately 770 hours of time-motion data and directly aligned with the workflow realities reported in earlier studies [6,7,36]. Together, these features can help bridge the gap highlighted by reviews that call for end-to-end, real-world evaluations beyond transcription accuracy alone [10,15]. The pattern of decreased active entry, waiting, and walking to the stationary ward computers, with slight increases in retrieval and review (see Table 3), also may show that mobile tools reduce “print-note-retype” loops [16].

Several mechanisms may influence the observed association between speech assistant use and reduced documentation time. First, this is in line with previous qualitative findings of Ferizaj and Neumann [19] that have shown fewer interruptions, less walking to the ward, shorter information-retrieval times, and higher overall satisfaction with the documentation system. In line with this, the mobile, as-you-go entry may have further reduced travel and queuing at ward PCs, cutting waiting, walking to the ward, and repeated logins. Second, domain-tuned speech and natural language processing (ie, models adapted to the nursing domain) may lower the friction of capturing structured information. This may reduce the need for interim notes and subsequent re-entry while also minimizing information loss and documentation errors. Third, workflow coupling at the bedside may decrease interruptions and handovers. The descriptive rise in information retrieval and review may reflect appropriate verification behavior within the mobile app. Usability in the good range [24] and stable acceptance [37] suggest the solution was well received with high intention-to-use and satisfaction, albeit with room for refinement.

### Limitations

This study has several limitations. The single-group pre-post design without a control condition limits causal inference and leaves room for workload fluctuations or co-occurring process changes as alternative explanations. According to reports from facility management, no other major documentation tools or workflow redesigns were introduced between *t*_0_ and *t*_1_, which reduces, but does not eliminate, the risk of confounding by concurrent initiatives. Participation was voluntary, so self-selection may have favored early adopters. Additionally, as facilities reported generally stable conditions, we did not collect shift-level workload indicators such as number of residents cared for or staffing ratios for each observed shift. Therefore, documentation time was not normalized for patient census or workload, and residual confounding by day-to-day fluctuations remains possible. Observations were conducted by multiple observers. Although observers underwent standardized training, joint pilot coding, and ongoing weekly calibration meetings, we did not calculate a formal interrater reliability coefficient (eg, Cohen κ) for the final field observations. This limits the extent to which we can quantify measurement consistency across observers. However, an exploratory sensitivity analysis including observer ID as a covariate did not materially change the estimated reduction in documentation time, suggesting that systematic between-observer differences are unlikely to account for the primary findings. Still, this exploratory analysis cannot rule out shared, potentially unconscious observer bias operating in the same direction across time points. Fourth, direct observation can itself change behavior (Hawthorne effect), which we sought to mitigate with standardized procedures but cannot exclude [25]. We did not systematically record time spent on disinfecting mobile devices unless it occurred in direct connection with documentation or monitor adherence to infection control protocols. If device disinfection is performed more consistently in routine use, the net time savings reported here may be overestimated. Documentation quality was captured perceptually rather than via objective measures such as assessing structured completeness or error rates that other work has examined in standardized tasks [12]. We did not conduct independent audits of documentation accuracy (eg, chart reviews, error checks). Reduced documentation time therefore cannot be directly linked to unchanged or improved documentation quality. Furthermore, dropouts were descriptively younger and less experienced than completers. This pattern may limit generalizability for adoption, since retention and continued engagement with the tool may differ by career stage, training status, or role-related workload. Finally, results reflect stationary long-term care in Germany, German-language ASR, and a specific EHR integration. Performance may differ with dialects, accents, terminology, and integration quality [15], and ongoing advances in speech technology may change effect sizes over time.

### Future Research

This study complements prior simulation and laboratory research and provides further evaluation of the potential effects of AI speech assistants in long-term care. To better inform workforce policy in the context of nursing shortages, future implementation research should examine whether documentation automation yields sustained workload relief, frees time for direct resident care, coordination, and recovery, and under which organizational and technical conditions these benefits can be realized at scale.

To strengthen causal inference and capture secular trends and learning effects, future studies should use cluster-randomized or stepped-wedge designs with extended follow-up. These evaluations should combine time-motion data with independent, objective assessments of documentation quality (eg, completeness indices, semantic accuracy ratings, and error rates) to determine whether time savings are achieved without compromising data integrity. In addition, studies should assess how saved time is reinvested and whether this translates into improvements in resident outcomes, psychosocial workload, perceived interruptions, staff satisfaction, and retention.

Methodologically, linking full-shift observation with EHR telemetry could quantify sustainability, diurnal patterns, and adoption trajectories. Additionally, further subgroup analyses could explore heterogeneity by accent/dialect, professional experience, unit characteristics, and integration context. Finally, economic evaluations should estimate the total cost of ownership and downstream effects on productivity, overtime, sick leave, and turnover to guide procurement and financing decisions for broader implementation.

### Conclusions

To the authors’ knowledge, this is the first study to report real-world time savings observed in association with the use of a mobile AI speech assistant in long-term care. In this longitudinal pre-post time-motion study conducted in 14 German long-term care facilities, implementation of a mobile, domain-specific speech documentation system into routine workflows was associated with substantially lower documentation time. Specifically, the mean documentation-related time decreased from about 54 minutes at baseline to approximately 39 minutes postimplementation. In the primary LMM with random intercepts for participants and facilities and fixed effects for time, age, gender, and baseline documentation type, the adjusted mean change was −15 minutes, corresponding to an adjusted 28% reduction relative to the baseline mean. This result remained consistent across sensitivity analyses and MNAR pattern-mixture scenarios. Task-level patterns indicated that time savings were primarily driven by less active documentation and note-taking, while reviewing entries and information retrieval showed small increases. This is consistent with a shift from producing text to verifying and retrieving information [19].

Secondary, exploratory self-report measures converged with the observational findings: participants reported fewer interruptions, lower perceived documentation burden, and higher satisfaction with the documentation system, while nursing workplace satisfaction did not change. Usability was rated as acceptable. Most participants experienced measurable time savings, with approximately one quarter reducing documentation time by more than about 24 minutes per shift. Given the single-group pre-post design and the reliance on self-reported indicators for documentation quality, these results should be interpreted as evidence of an association between implementation and reduced documentation time rather than as causal proof or as objective evidence of changes in documentation quality.

## Supplementary material

10.2196/86078 Multimedia Appendix 1 Definition of task categories for observations at *t*_0_ and *t*_1_.

10.2196/86078 Multimedia Appendix 2 Self-developed questionnaire for assessment at *t*_0_ and *t*_1_.

10.2196/86078 Multimedia Appendix 3 Statistical analysis rationale and detailed methods.

10.2196/86078 Multimedia Appendix 4 Bayesian supplementary modeling and analysis.

10.2196/86078 Multimedia Appendix 5 Results of exploratory sensitivity analyses.

## Abbreviations

AI: artificial intelligence
ASR: automatic speech recognition
EHR: electronic health record
LMM: linear mixed-effects model
MNAR: missing not at random
NWSQ: Nursing Workplace Satisfaction Questionnaire
PYSA: “Pflegedokumentation mit Hybridem Sprachassistenten” (project title)
STROBE: Strengthening the Reporting of Observational Studies in Epidemiology
SUS: system usability scale
*t*_0_: baseline measurement
*t*_1_: postimplementation measurement
