# Supplementary material for: Time Savings Through an AI Speech Assistant for Nursing Documentation: Pre-Post Time-Motion Study in German Long-Term Care
Source: J Med Internet Res. 2026 Apr 8;28:e86078. doi: 10.2196/86078 (PMC13061367; doi:10.2196/86078)
Supplement: Multimedia Appendix 1 [file jmir-v28-e86078-s001.docx]

**Multimedia Appendix 1**

**Definition of Task Categories for Observations at *t*_0_ and *t*_1_**

| **Table S1.** Task categories and their definitions for observations before the implementation of voize (t_0_) | |
| --- | --- |
| **Task (t_0_)** | **Description** |
| Documentation at the PC/on paper | A nurse enters documentation directly into the electronic record at a ward computer or writing documentation on paper: signing off on interventions, writing nursing reports, filling out protocols such as drinking or nutrition. Documentation of wound progress, etc. |
| Informing on the PC/file | A nurse retrieves information relating to residents from the documentation system (PC or paper file). This information is passed on to other nursing and care staff and doctors, or is required by the nurse for their own nursing tasks. |
| Booting up and logging into the PC | A nurse starts the PC, logs in to the PC with the password, and/or logs in to the documentation system with the password. |
| Waiting for PC | A nurse waits for a workstation to become available. No other tasks are performed during this time. |
| Making notes | A nurse writes temporary notes, such as vital signs or fluid intake for later documentation. |
| Walking to the computer | A nurse walks to the ward PC with the aim of documenting or retrieving information about residents. Walking that occurs while documentation is being performed (eg, dictating with the mobile speech assistant while moving at t_1_) is not coded here but counted only as documentation time. |
| Reviewing entries | A nurse reviews nursing documentation entries, either their own records or those of colleagues such as nursing assistants or trainees. |

| **Table S2.** Additional task categories measured at t_1_, in addition to those defined at t_0_ | |
| --- | --- |
| **Task (t_1_)** | **Description** |
| Documentation in voize | A nurse takes the smartphone out of their pocket, unlocks it, and documents measures in voize using voice input and/or signs them manually and/or documents them using freehand mode. The smartphone is locked again. |
| Informing in voize | A nurse takes her smartphone out of their pocket, unlocks it, and retrieves information about residents manually or via voice input. |
