# Supplementary material for: Time Savings Through an AI Speech Assistant for Nursing Documentation: Pre-Post Time-Motion Study in German Long-Term Care
Source: J Med Internet Res. 2026 Apr 8;28:e86078. doi: 10.2196/86078 (PMC13061367; doi:10.2196/86078)
Supplement: Multimedia Appendix 2 [file jmir-v28-e86078-s002.docx]

**Multimedia Appendix 2**

**Self-Developed Questionnaire for Assessment at *t*_0_ and *t*_1_**

| **Table S1.** Constructs and self-developed items for t_0_ assessment | |
| --- | --- |
| **Assessment Domains** | **Item** |
| Satisfaction | How satisfied are you with the current documentation process and system?  What is your current mode of documentation? |
|  | Do you think it's a good idea to introduce voize for care documentation? |
|  | How likely are you to use voize regularly for care documentation? |
| Perceived documentation quality and efficiency | voize will improve the quality of my care documentation.  voize will save me time on care documentation.  My current nursing documentation is of high quality.  My current nursing documentation is complete and comprehensive.  The current nursing documentation process is time-consuming.  I am frequently interrupted while performing nursing tasks.  Currently, I need approximately the following for the entire care documentation per shift: {{time_in_minutes}} |
|  | What work experiences do you expect from the introduction of voize? |

| **Table S2.** Constructs and self-developed items for t_1_ assessment | |
| --- | --- |
| **Assessment Domains** | **Item** |
| Satisfaction | How long have you been using voize? |
|  | How often do you use voize per week? |
|  | Which type of care documentation do you currently use most frequently? |
|  | How satisfied are you overall with voize? |
|  | Was it a good idea to introduce voize for care documentation? |
| Perceived documentation quality and efficiency | voize has improved the quality of care documentation.  voize saves me time when searching for information.  I plan to continue using voize for care documentation in the future.  With voize, my documentation entries are complete and comprehensive.  Care documentation with voize is time-consuming.  My current nursing documentation is of high quality.  I am frequently interrupted while performing nursing tasks.  With the help of voize, I save time on care documentation.  With voize, I need about the following for all care documentation per shift: {{time_in_minutes}} |
