# Supplementary material for: Time Savings Through an AI Speech Assistant for Nursing Documentation: Pre-Post Time-Motion Study in German Long-Term Care
Source: J Med Internet Res. 2026 Apr 8;28:e86078. doi: 10.2196/86078 (PMC13061367; doi:10.2196/86078)
Supplement: Multimedia Appendix 3 [file jmir-v28-e86078-s003.docx]

# Multimedia Appendix 3

# Statistical Analysis Rationale and Detailed Methods

## Data structure and estimand

The primary endpoint (total documentation time per observed morning shift in minutes) was measured repeatedly within participants at baseline (t_0_) and post-implementation (t_1_). Observations were therefore clustered at two levels: repeated measurements nested within nurses, and nurses nested within long-term care facilities. To obtain valid inference under this dependency structure and to use all available repeated-measures information, we analyzed the primary endpoint with a linear mixed-effects model (LMM) including random intercepts for participant and facility. The primary estimand was the covariate-adjusted mean change in total documentation time from baseline to post-implementation (t_1_-t_0_), expressed in minutes.

## Primary mixed model specification

We fit the primary LMM in long format (one row per participant per time point). Time was coded as 0 at baseline and 1 at post-implementation. Thus, the fixed time effect directly represents the adjusted mean change (t_1_-t_0_). Additional fixed effects covariates were baseline documentation type (categorical), gender (coded as binary and mean-centered), and age (continuous, centered). Random intercepts were included for participants and facilities to model within-person correlation and facility-level clustering. With two measurements, this specification corresponds to a mixed model for repeated measures in which baseline and follow-up are jointly modeled as outcomes, so that participants with only one observed time point can still contribute information via the likelihood. For analyses fitted to a single dataset, models were estimated using restricted maximum likelihood (REML), and small-sample inference for fixed effects used Kenward-Roger degrees of freedom [[1]](https://www.zotero.org/google-docs/?PuqJco). All tests were two-sided at α=.05.

## Missing Data Handling

Missingness in the primary outcome occurred only at t_1_ due to dropouts. Complete-case analysis is unbiased under MCAR and may be biased under MAR unless additional conditions hold [[2]](https://www.zotero.org/google-docs/?nibBzB). Under a MAR mechanism, likelihood-based mixed models can provide valid inference without explicit outcome imputation by using all available repeated measures [[2,3]](https://www.zotero.org/google-docs/?4gJ0aJ). Accordingly, the primary analysis relied on the likelihood-based LMM, with additional sensitivity analyses described below.

### **Multiple imputation under MAR and pooling**

Furthermore, to assess stability of missing outcome estimates, we performed multiple imputation with 40 datasets. Imputation used tree-based predictive mean matching in miceforest [[4]](https://www.zotero.org/google-docs/?EpKCu0) (400 trees, 10 donors, up to 10 iterations). To preserve the clustered data structure, the imputation model included, in addition to baseline and all (primary and secondary) endpoint variables, covariates and study design factors such as documentation type at baseline, facility, age, gender, and observer. Across the multiply imputed datasets, we refit the same primary LMM and pooled fixed-effect estimates using Rubin’s rules, combining within- and between-imputation uncertainty [[2]](https://www.zotero.org/google-docs/?ces90K) and small-sample inference after pooling used the Barnard-Rubin degrees-of-freedom adjustment [[5]](https://www.zotero.org/google-docs/?eGVJ75). As an additional simple robustness check, we refit the primary LMM to complete cases.

## Alternative LMM specifications

Because time-motion measurements were recorded by trained observers, we added an observer random intercept to account for systematic between-observer differences in recorded documentation time. Additionally, we estimated cluster-robust (CR2) standard errors clustered by facility as an additional check against residual facility-level misspecification of the working correlation structure.

To evaluate whether the time effect differed by baseline characteristics, we fit sequential exploratory models including time-by-covariate interactions for age, gender, and baseline documentation type. We acknowledge that subgroup tests are typically underpowered, especially in smaller samples where baseline characteristics split the data into even smaller contrasts. These interaction models must be interpreted cautiously given limited power. Still, we aimed to align with guidance noting that inclusion of covariate-by-time interactions can matter for repeated measures and can help to maintain power gains [[6]](https://www.zotero.org/google-docs/?NSS05M).

## Missing-Not-At-Random pattern-mixture sensitivity analyses

Because MAR is not directly testable, we conducted Missing Not At Random (MNAR) sensitivity analyses using a pattern-mixture framework implemented via controlled modifications of imputed values across the 40 datasets generated by miceforest. Specifically, we identified participants with missing post-implementation outcomes in the original dataset and, starting from the MAR-imputed datasets, systematically altered their imputed post values to represent progressively more conservative departures from MAR. Three MNAR scenarios were evaluated:

1. MAR + δ adjustment (δ shift; post := post + δ)
   This assumes that, among those missing at t_1_, the true documentation time would be higher than the MAR-imputed value by δ minutes (i.e., less improvement than imputed). Positive δ therefore attenuates the estimated reduction.
2. Jump-to-baseline (post := baseline)
   This assumes no improvement among those missing at t_1_. In this case, their post-implementation documentation time would “jump” back to their baseline values.
3. Jump-to-baseline + δ (post := baseline + δ)
   This assumes deterioration among those missing at t1 by δ minutes relative to baseline.

We evaluated δ on a prespecified grid (0-45 minutes in 1-minute steps). For each δ and scenario, the primary LMM was refit on each imputed dataset and pooled according to Rubin’s rules with Barnard-Rubin degrees of freedom. Tipping points were defined as the smallest δ at which the pooled time effect became non-significant (*P*≥.05).

## ANCOVA-of-change as an Exploratory Complementary Analysis

As a complementary robustness check, we estimated an ANCOVA-of-change on the individual change score (t_1_-t_0_) for total documentation time. Specifically, we modeled the change score as a linear function of centered baseline documentation time, centered age, baseline documentation type (paper vs. hybrid vs. PC only), facility, and gender (coded as a binary and mean-centered). Therefore, the model intercept estimates the adjusted mean change in documentation time for a nurse with average baseline documentation time, age, and gender, in the reference categories of the categorical covariates baseline documentation type and facility. Baseline adjustment is commonly recommended in pre-post and trial settings because it can improve precision when baseline values are prognostic [[6]](https://www.zotero.org/google-docs/?2nTyEI).

## Multiple imputation and pooling for ANCOVA

We repeated the ANCOVA-of-change analysis using the same m=40 MAR imputations and pooled estimates using Rubin’s rules with Barnard-Rubin degrees of freedom [[5]](https://www.zotero.org/google-docs/?a29qXp). We repeated the ANCOVA-of-change estimand under the exact same MNAR departures as the LMM sensitivity analyses. If no tipping point was identified within the prespecified δ=0-45-minute grid, δ was extended beyond 45 minutes until a tipping point was found.

**Secondary outcome modelling**

All secondary outcomes, including questionnaire scale and single items on a Likert scale, were analyzed exploratorily as within‑subject changes from baseline to post-implementation. For each outcome, we computed an individual change score (t_1_-t_0_) and estimated the mean change in the original units of the respective scale. Inference was obtained by fitting an intercept-only linear model to the change scores and testing whether the mean change differed from zero. This targets the same mean-change point estimate as the classical paired Student’s *t*-test, that is, a one-sample test of the paired differences. However, uncertainty was quantified using HC3 robust standard errors to construct 95% confidence intervals and two-sided *P*-values.

Analyses were conducted using multiple imputation with the same specification as in the MAR sensitivity analysis of the primary LMM with 40 multiply imputed datasets. The same change-score model was fit within each imputed dataset and pooled using Rubin’s rules, with the Barnard-Rubin small-sample degrees-of-freedom adjustment [5] for pooled *t*-based inference. To control the family-wise error rate across the 13 secondary outcomes, *p*-values were adjusted using Holm’s sequentially rejective Bonferroni procedure, and Holm-adjusted *P*-values are reported.

**References**

[1.](https://www.zotero.org/google-docs/?BkhOVQ)  [Kenward MG, Roger JH. Small sample inference for fixed effects from restricted maximum likelihood. Biometrics JSTOR; 1997;983–997.](https://www.zotero.org/google-docs/?BkhOVQ)

[2.](https://www.zotero.org/google-docs/?BkhOVQ)  [Rubin DB. Inference and missing data. Biometrika Oxford University Press; 1976;63(3):581–592.](https://www.zotero.org/google-docs/?BkhOVQ)

[3.](https://www.zotero.org/google-docs/?BkhOVQ)  [Little RJ, D’agostino R, Cohen ML, Dickersin K, Emerson SS, Farrar JT, Frangakis C, Hogan JW, Molenberghs G, Murphy SA. The prevention and treatment of missing data in clinical trials. N Engl J Med Mass Medical Soc; 2012;367(14):1355–1360.](https://www.zotero.org/google-docs/?BkhOVQ)

[4.](https://www.zotero.org/google-docs/?BkhOVQ)  [Wilson S. Miceforest: Missing Value Imputation Using LightGBM (5.6. 3)[Python; MacOS, Microsoft:: Windows, OS Independent]. 2022.](https://www.zotero.org/google-docs/?BkhOVQ)

[5.](https://www.zotero.org/google-docs/?BkhOVQ)  [Barnard J, Rubin DB. Miscellanea. Small-sample degrees of freedom with multiple imputation. Biometrika Oxford University Press; 1999;86(4):948–955.](https://www.zotero.org/google-docs/?BkhOVQ)

[6.](https://www.zotero.org/google-docs/?BkhOVQ)  [Schuler A. Mixed models for repeated measures should include time-by-covariate interactions to assure power gains and robustness against dropout bias relative to complete-case ANCOVA. Ther Innov Regul Sci Springer; 2022;56(1):145–154.](https://www.zotero.org/google-docs/?BkhOVQ)
