# Supplementary material for: Time Savings Through an AI Speech Assistant for Nursing Documentation: Pre-Post Time-Motion Study in German Long-Term Care
Source: J Med Internet Res. 2026 Apr 8;28:e86078. doi: 10.2196/86078 (PMC13061367; doi:10.2196/86078)
Supplement: Multimedia Appendix 4 [file jmir-v28-e86078-s004.docx]

# Multimedia Appendix 4

# Bayesian Supplementary Modelling and Analysis

## Methods

## Overview and rationale

The data have a hierarchical structure: repeated measurements (baseline and post-implementation) are nested within nurses, and nurses are nested within care facilities. This induces within-nurse correlation over time and clustering within facilities. Therefore, the primary analysis uses a frequentist linear mixed-effects model (LMM) with random intercepts for nurses and facilities, estimated in a frequentist framework. In addition, we conducted a complementary Bayesian analysis to triangulate the frequentist results and to align with the “new statistics” paradigm, which emphasizes estimation, uncertainty, and cumulative evidence rather than dichotomous decisions based on null-hypothesis testing alone [[1]](https://www.zotero.org/google-docs/?HU2zjx).

We chose complementary Bayesian modelling as a robustness and enrichment strategy for three reasons. First, Bayesian inference yields interpretable probability statements about clinically relevant quantities. In this study, this could refer to the probability that documentation time decreased. Second, Bayesian hierarchical modelling naturally propagates uncertainty across levels (nurse and facility) and across multiple outcomes through partial pooling. Third, the Bayesian framework allowed joint imputation of missing outcomes within the model and Missing-Not-At-Random (MNAR) sensitivity analyses using pattern-mixture departures, implemented as δ-adjustments applied to posterior draws of missing post values [[1,2]](https://www.zotero.org/google-docs/?5c8IKT).

## Bayesian Inference for the Primary Model

Bayesian inference updates prior beliefs with observed data through Bayes’ theorem. In applied terms, a Bayesian model combines a likelihood that encodes the assumed data-generating process and priors that encode regularizing information about plausible parameter values, producing a posterior distribution that quantifies uncertainty after observing the data [[3]](https://www.zotero.org/google-docs/?IEXczv). This posterior distribution supports questions that can be directly aligned with applied decision-making, such as in our case: (1) What is the probability that total documentation time decreased after the implementation of a speech-based AI assistant?

## Bayesian preprocessing and scaling

To place effects on comparable scales and to make weakly informative priors meaningful across outcomes, continuous predictors and all secondary outcomes were scaled by dividing by 2 standard deviations (Gelman’s 2-SD scaling [[6]](https://www.zotero.org/google-docs/?lAeVXw)). With this scaling, a coefficient corresponds approximately to moving from a “typical low” to a “typical high” value, facilitating interpretability and prior selection/calibration. 2-SD scaled variables were back-transformed for reporting in original units.

## Glossary and Core Bayesian Concepts Applied

***Prior and posterior predictive checks.***

Prior predictive checks assess whether your priors and likelihood imply plausible data before fitting the model. Simulated parameters are drawn from the prior, replicated outcomes are generated from the likelihood, and one (often visually) inspect whether the implied ranges and shapes are reasonable (e.g., in our case, no negative documentation time and no extreme values). In contrast, posterior predictive checks assess model adequacy after fitting by simulating replicated datasets from the posterior predictive distribution and visually comparing them to the observed data (e.g., overlaid densities; checking whether the model reproduces key features such as location, spread, skewness, and outliers) [3,10].

***Global-local shrinkage via regularized horseshoe.***

Because we have a small sample and the secondary outcomes are exploratory and numerous, we used a regularized horseshoe prior for the vector of secondary post-baseline changes. This prior allows a small number of outcomes to show larger changes while shrinking most effects toward zero, reducing overfitting and mitigating false positives in multivariate settings [[5]](https://www.zotero.org/google-docs/?3aUNcc).

***Hierarchical priors and partial pooling.***

For multiple secondary outcomes, we used hierarchical priors that shrink outcome-specific changes toward a common mean when the data are weak, improving stability in small samples.

### Posterior distribution, highest density interval, and region of practical equivalence.

For each estimand (e.g., the post-baseline change in documentation time), Bayesian inference yields a posterior distribution, that is, a distribution of plausible parameter values given the model, priors, and observed data. We summarize posteriors using posterior means, posterior SDs, and 94% highest density interval (HDIs; the default interval probability in ArviZ [4]). A 94% HDI is the narrowest interval containing 94% of the posterior mass. Thus, values inside the HDI are more plausible than values outside, given the model and priors.

To complement interval estimates with a notion of practical relevance, we defined a region of practical equivalence (ROPE) around the null value. The ROPE is an interval of effect sizes considered practically negligible for current purposes. We report Pr(|Δ| ≤ ROPE) as the posterior probability that the change is practically negligible, and (when a direction is substantively meaningful) Pr(Δ<−ROPE) or Pr(Δ>ROPE) as the posterior probability of a meaningfully negative or positive change beyond the ROPE.

### Primary outcome model.

The primary outcome (total documentation time per morning shift in minutes) was modeled using a lognormal likelihood with nurse-level and facility-level random intercepts. Documentation time was strictly positive and the log scale provides a natural multiplicative interpretation, where effects map directly to percent changes. Baseline and post values were modeled jointly as repeated measures, meaning that baseline was treated as an outcome and not reused as a covariate.

Time was coded as -0.5 for baseline and +0.5 for post. Thus, the time coefficient β_time_ directly represents the difference in log means. Throughout, we define Δ = post-baseline on the minutes scale. Therefore, reductions correspond to Δ < 0. Adjustment covariates (age, gender, baseline documentation type) entered the model as main effects with no interaction effects. A ROPE of 0.1 SD was set for the primary outcome.

A representation of the primary likelihood is:

[
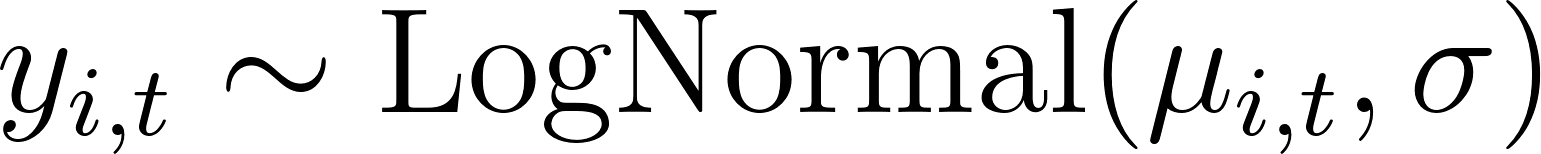
](https://www.codecogs.com/eqnedit.php?latex=%5C%5C%5C%5C%20y_%7Bi%2Ct%7D%20%5Csim%20%5Cmathrm%7BLogNormal%7D(%5Cmu_%7Bi%2Ct%7D%2C%20%5Csigma)%5C%5C%5C%5C%20#0)

[
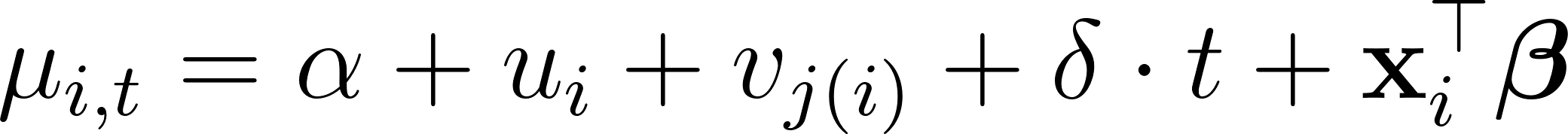
](https://www.codecogs.com/eqnedit.php?latex=%5C%5C%5C%5C%20%5Cmu_%7Bi%2Ct%7D%20%3D%20%5Calpha%20%2B%20u_i%20%2B%20v_%7Bj(i)%7D%20%2B%20%5Cdelta%20%5Ccdot%20t%20%2B%20%5Cmathbf%7Bx%7D_i%5E%5Ctop%20%5Cboldsymbol%7B%5Cbeta%7D%5C%5C%5C%5C%20#0)

where [
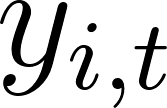
](https://www.codecogs.com/eqnedit.php?latex=y_%7Bi%2Ct%7D#0) is documentation time for nurse [
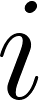
](https://www.codecogs.com/eqnedit.php?latex=i#0) at time [
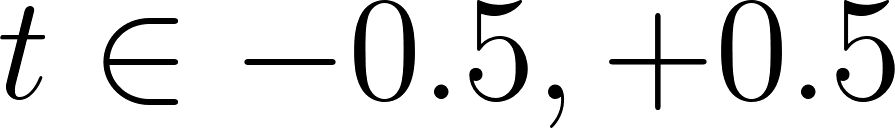
](https://www.codecogs.com/eqnedit.php?latex=t%20%5Cin%20%7B-0.5%2C%20%2B0.5%7D#0), [
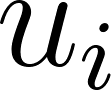
](https://www.codecogs.com/eqnedit.php?latex=u_i#0) is a nurse random intercept, [
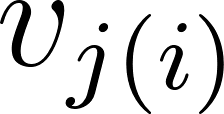
](https://www.codecogs.com/eqnedit.php?latex=v_%7Bj(i)%7D#0) is a facility random intercept, and [
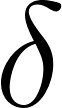
](https://www.codecogs.com/eqnedit.php?latex=%5Cdelta#0) is the primary post-baseline change on the log scale. For interpretability, we reported posterior summaries of derived quantities on the minutes scale, including posterior means of the baseline and post marginal means, the posterior distribution of the mean change Δ=post-baseline in minutes, and the posterior distribution of the percent reduction defined as 100 × (1 − post/baseline), computed from the posterior draws of the model-implied marginal means.

### Secondary outcome model.

Secondary outcomes were modeled jointly with a robust Student-*t* likelihood (ν fixed at 4) on the 2-SD standardized scale to reduce sensitivity to outliers and non-normality. For each secondary outcome [
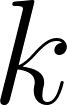
](https://www.codecogs.com/eqnedit.php?latex=k#0), we modeled baseline and post as repeated measures with an outcome-specific post-baseline change parameter [
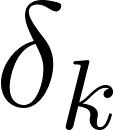
](https://www.codecogs.com/eqnedit.php?latex=%5Cdelta_k#0) as well as a random facility intercept, including the covariates age, gender, and baseline documentation type. To account for dependence across outcomes within nurses, the main specification used a parsimonious rank-1 latent factor structure for subject-level random effects, inducing correlations across secondary outcomes. To demonstrate robustness of this modelling choice, we additionally fit:

1. Rank-2 and rank-3 factor models. Two- and three-factor structures relaxed the rank-1 assumption while remaining parsimonious given n=52.
2. Multiplicative gamma process (MGP) shrinkage factor model. An overcomplete factor model with multiplicative gamma shrinkage allowed the data to regularize the effective number of factors, providing a flexible but regularized alternative [[8]](https://www.zotero.org/google-docs/?6F6fDJ)
3. Full covariance model. A full multivariate subject random intercept covariance matrix was estimated using an LKJ prior with [
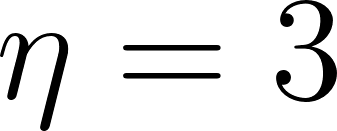
](https://www.codecogs.com/eqnedit.php?latex=%5Ceta%3D3#0) to mildly favor correlations near zero [[7]](https://www.zotero.org/google-docs/?tZA9pq).

## Prior Selection

All priors were weakly informative and chosen to stabilize estimation in small samples while remaining compatible with plausible effect sizes on the scaled outcome scale. For scale parameters (standard deviations), we used half-normal or half-Student-*t* priors consistent with recommendations for hierarchical models [[9]](https://www.zotero.org/google-docs/?I5gmTy).

### Primary outcome priors.

Documentation time was modeled with a lognormal likelihood and nurse- and facility-level random intercepts. The intercept was centered on the log of the observed median minutes to aid calibration (SD 1.0). The primary time effect [
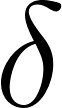
](https://www.codecogs.com/eqnedit.php?latex=%5Cdelta#0) used a zero-centered normal prior with moderate scale (base: SD 0.25 on the log scale; skeptical variant: SD 0.18). Random-intercept standard deviations for nurse and facility used half-normal priors (0.30 for nurses and 0.25 for facilities). The residual log-scale dispersion used a half-Student-*t* prior (ν=4, scale 0.5).

### Secondary outcomes priors.

Secondary intercepts had normal priors centred at 0 (SD 0.5) and residual scales had half-normal priors (scale 0.5) on the standardized scale. For the rank-1 subject factor, the subject-factor SD used a half-normal prior (scale 0.5), and loadings had standard normal priors with the first loading fixed to 1 for identifiability. Additionally, we considered three regularizing families of priors for the vector of secondary changes [
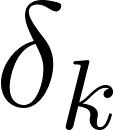
](https://www.codecogs.com/eqnedit.php?latex=%5Cdelta_k#0):

1. Hierarchical normal prior (“base”). Each [
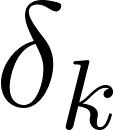
](https://www.codecogs.com/eqnedit.php?latex=%5Cdelta_k#0) was drawn from a common normal distribution with an estimated mean and dispersion, supporting partial pooling across outcomes. Across-outcome mean change was normal centered at 0 (SD 0.15) and between-outcome heterogeneity half-normal (scale 0.25).
2. Hierarchical normal prior (“skeptical”). The same structure as hierarchical normal prior (“base”) with tighter hyperpriors (mean SD 0.10; heterogeneity scale 0.20).
3. Regularized horseshoe prior. To reflect that many exploratory secondary changes are likely near zero, while allowing a few to be larger if supported by the data, we used a regularized horseshoe prior. This prior uses a global-local shrinkage prior with an explicit slab that prevents unrealistically extreme coefficients when data are weak. We used:
   - A global shrinkage parameter that controls overall sparsity: [
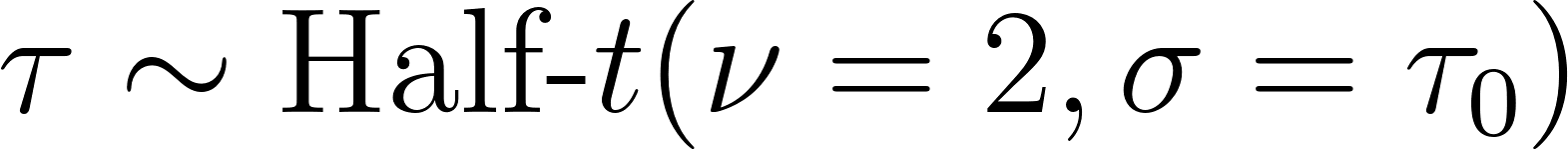
](https://www.codecogs.com/eqnedit.php?latex=%5Ctau%20%5Csim%20%5Ctext%7BHalf-%7Dt(%5Cnu%3D2%2C%5Csigma%3D%5Ctau_0)#0), where [
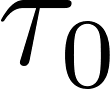
](https://www.codecogs.com/eqnedit.php?latex=%5Ctau_0#0) is set using an expected number of nonzero effects heuristic with [
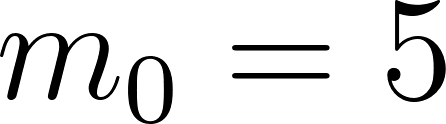
](https://www.codecogs.com/eqnedit.php?latex=m_0%3D5#0) (capped at [
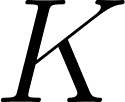
](https://www.codecogs.com/eqnedit.php?latex=K#0)) and scaled by [
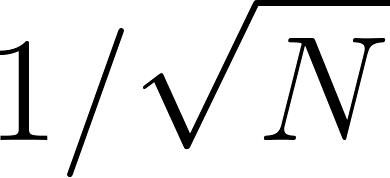
](https://www.codecogs.com/eqnedit.php?latex=1%2F%5Csqrt%7BN%7D#0).
   - Local shrinkage parameters that allow specific outcomes to deviate when supported by data: [
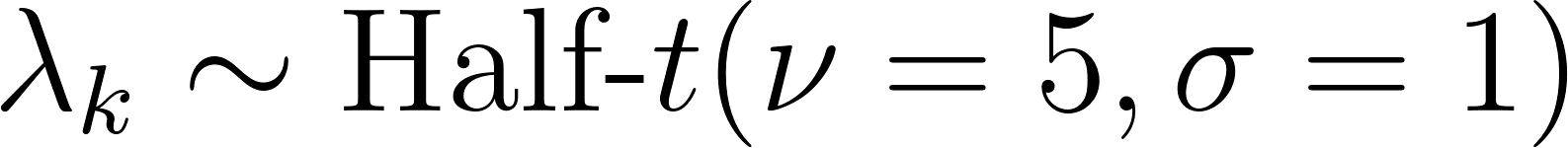
](https://www.codecogs.com/eqnedit.php?latex=%5Clambda_k%20%5Csim%20%5Ctext%7BHalf-%7Dt(%5Cnu%3D5%2C%5Csigma%3D1)#0) for [
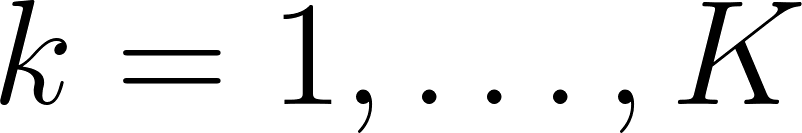
](https://www.codecogs.com/eqnedit.php?latex=k%3D1%2C%5Cdots%2CK#0).
   - A regularization term that prevents extreme coefficients when data are weak: [
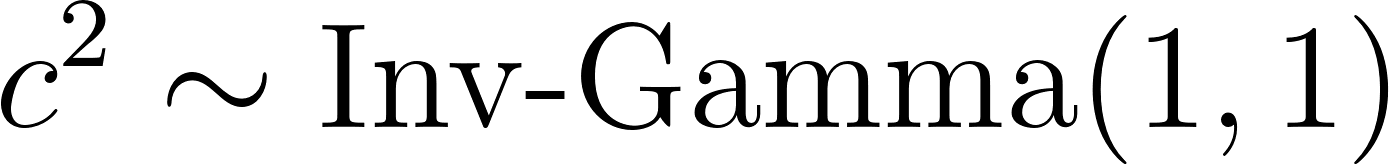
](https://www.codecogs.com/eqnedit.php?latex=c%5E2%20%5Csim%20%5Ctext%7BInv-Gamma%7D(1%2C1)#0).

## Missing data handling and Missing-Not-At-Random sensitivity analyses

### Joint imputation within the Bayesian model.

When the primary or secondary outcomes contained missing values, PyMC treated them as latent variables and sampled them from their posterior distribution conditional on the observed data and model [10]. This yields joint imputation that is coherent with the assumed likelihood and priors and that propagates imputation uncertainty into all posterior summaries.

### Missing-Not-At-Random pattern-mixture departures for the primary outcome.

Because dropouts occurred between baseline and post-implementation (8/52, 15.4%), we assessed how extreme MNAR deviations would need to be to overturn the conclusion that documentation time decreased. We implemented two tipping-point scenarios for the missing post primary outcomes:

1. MAR + δ adjustment (δ shift; post := post + δ). For missing post values, we added a constant δ minutes and re-evaluated the posterior probability of a reduction.
2. Jump-to-baseline + δ (post := baseline + δ). For missing post values, we first set post equal to the participant’s baseline value indicating no improvement, then added δ minutes.

We searched δ in a grid from 0 to 45 minutes and identified whether Pr(Δ<0) would drop below a prespecified threshold of 0.95. This Bayesian tipping-point procedure differs from frequentist tipping-point analyses in that δ-adjustments are applied directly to posterior draws of missing post values rather than repeatedly refitting the full model under shifted imputations.

## Computation and diagnostics

All Bayesian models were estimated using PyMC [[10]](https://www.zotero.org/google-docs/?Kjs0Ch) with Hamiltonian Monte Carlo via the No-U-Turn Sampler. The primary and secondary outcomes as well as their missing values were estimated within a joint Bayesian model. The base model was sampled with 4 chains, 1500 tuning iterations, and 1000 posterior draws per chain. Sensitivity models used 1200 tuning iterations and 800 posterior draws per chain. We used a target acceptance rate of 0.97 and maximum tree depth of 12. Convergence and sampling quality were assessed using the number of divergences, the fraction of maximum tree depth hits, effective sample sizes, and [
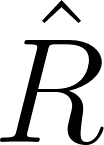
](https://www.codecogs.com/eqnedit.php?latex=%5Chat%7BR%7D#0). Additionally, we performed both prior and posterior predictive checks for the primary outcome and a subset of secondary outcomes. These checks showed adequate fit.

## Results

### Primary outcome.

In the Bayesian repeated-measures lognormal model, the posterior mean documentation time decreased from 54.23 (posterior SD 2.85) minutes at baseline to 40.02 (posterior SD 2.33) minutes post-implementation. The posterior mean change was (Δ as post-baseline) −14.20 (posterior SD 3.44) minutes (94% HDI −20.89 to −7.74). The posterior mean percent reduction was 26.0% (posterior SD 5.4%; 94% HDI 15.9%–36.3%). Furthermore, the probability that the posterior change lays within the ROPE was Pr(|Δ| ≤ ROPE) = 0.000.

**Table S1.** Primary outcome posterior summaries across Bayesian sensitivity models (minutes and percent).

| **Model** | **Baseline mean, minutes (SD)** | **Post mean, minutes (SD)** | **Mean Change (post-baseline), minutes (SD)** | **94% HDI, minutes** | **Pr(Δ<0)** | **Mean percent Reduction (SD)** | **94% HDI, %** |
| --- | --- | --- | --- | --- | --- | --- | --- |
| Hierarchical Base priors model | 54.19 (2.72) | 40.05 (2.31) | -14.13 (3.40) | -19.99 to  -7.18 | 1.000 | 25.9 (5.4) | 15.3-35.3 |
| Hierarchical Skeptical priors model | 53.69 (2.66) | 40.57 (2.39) | -13.12 (3.34) | -19.66 to -7.08 | 1.000 | 24.3 (5.5) | 14.1-34.1 |
| Horseshoe prior model | 54.23 (2.85) | 40.02 (2.33) | -14.20 (3.44) | -20.89 to -7.74 | 1.000 | 26.0 (5.4) | 15.9-36.3 |

### Note. Table entries are posterior means. Values in parentheses are posterior SDs indicating the uncertainty of the estimated mean or change. Baseline and post values across primary and secondary outcomes were modeled jointly as repeated measures, and missing outcomes were imputed within the Bayesian model.

### Secondary outcomes.

Secondary outcomes were modeled jointly and are reported as posterior mean changes (post minus pre) in original units, alongside standardized changes on the 2-SD scale. We additionally report Pr(Δ<0), Pr(Δ>0), and Pr(|Δ|≤ROPE), where the ROPE was defined as ±0.05 on the 2-SD scale corresponding to approximately ±0.10 SD and Δ denotes the post-baseline change. The horseshoe prior analysis served as the main Bayesian analysis for secondary changes because it regularizes the family of exploratory effects and increases the probability mass near zero for outcomes with weak evidence. Because gains in out-of-sample predictive performance were negligible with more complex covariance structures, we report the rank-1 factor model with the regularized horseshoe prior on secondary changes as the main specification for parsimony and stability.

**Table S2**. Secondary outcomes of the main Bayesian model (horseshoe prior on secondary changes with a rank-1 subject factor).

| **Outcome (Score range)** | **Mean change (post–pre), original units (SD)** | **94% HDI, original units** | **Mean change (post–pre), 2-SD units (SD)** | **94% HDI, 2-SD units** | **Pr(Δ<0)** | **Pr(Δ>0)** | **Pr(\|Δ\| ≤ ROPE)^a^** |
| --- | --- | --- | --- | --- | --- | --- | --- |
| NWSQ: Intrinsic (Range: 6–30) | -0.16 (0.56) | -1.37 to 0.83 | -0.02 (0.07) | -0.16 to 0.10 | 0.635 | 0.365 | 0.621 |
| NWSQ: Extrinsic (Range: 5–25) | 0.40 (0.44) | -0.32 to 1.28 | 0.07 (0.08) | -0.06 to 0.23 | 0.181 | 0.819 | 0.423 |
| NWSQ: Relational (Range: 4–20) | -0.28 (0.36) | -1.05 to 0.31 | -0.05 (0.07) | -0.19 to 0.06 | 0.774 | 0.226 | 0.506 |
| Satisfaction with Documentation system (Range: 1–10) | 1.78 (0.43) | 0.99 to 2.61 | 0.40 (0.10) | 0.22 to 0.59 | 0.000 | 1.000 | 0.000 |
| Implementation as a good idea (Range: 1–10) | -0.21 (0.30) | -0.82 to 0.31 | -0.05 (0.07) | -0.20 to 0.08 | 0.750 | 0.250 | 0.508 |
| Intention to use (Range: 1–10) | -0.20 (0.27) | -0.73 to 0.26 | -0.05 (0.07) | -0.19 to 0.07 | 0.768 | 0.232 | 0.507 |
| Expected quality improvement (Range: 1–5) | -0.20 (0.15) | -0.48 to 0.06 | -0.12 (0.09) | -0.29 to 0.04 | 0.914 | 0.086 | 0.243 |
| Expected Time Savings (Range: 1–5) | -0.11 (0.13) | -0.37 to 0.09 | -0.07 (0.08) | -0.24 to 0.06 | 0.807 | 0.193 | 0.422 |
| Documentation is complete (Range: 1–5) | -0.34 (0.22) | -0.72 to 0.04 | -0.17 (0.11) | -0.38 to 0.02 | 0.945 | 0.055 | 0.148 |
| High Documentation Quality (Range: 1–5) | -0.25 (0.16) | -0.53 to 0.03 | -0.15 (0.10) | -0.32 to 0.02 | 0.951 | 0.049 | 0.167 |
| Documentation is Time-consuming (Range: 1–5) | -1.65 (0.20) | -2.02 to -1.28 | -0.70 (0.08) | -0.86 to -0.54 | 1.000 | 0.000 | 0.000 |
| Interruptions in daily work (Range: 1–5) | -0.44 (0.19) | -0.78 to -0.05 | -0.24 (0.10) | -0.42 to -0.03 | 0.993 | 0.007 | 0.037 |
| Self-reported documentation time (in minutes) | -24.85 (7.30) | -38.54 to -10.93 | -0.31 (0.09) | -0.49 to -0.14 | 0.999 | 0.001 | 0.005 |

^a^The ROPE was ±0.05 on the 2-SD scale (approximately ±0.10 SD).

### Note. Table entries are posterior means. Values in parentheses are posterior SDs indicating the uncertainty of the estimated mean or change.

### Sensitivity to prior choices for secondary outcomes.

Comparing the hierarchical-normal prior models (base and skeptical) with the horseshoe prior model showed that large, consistent effects remained essentially unchanged, whereas smaller effects were more strongly shrunk toward zero under the horseshoe prior. For example, the increase in satisfaction with the documentation system remained large and unambiguous across priors (posterior means 1.56-1.78; all 94% HDIs excluding 0), and the decrease in perceived documentation time burden remained large (posterior means -1.55 to -1.65; all 94% HDIs excluding 0). In contrast, outcomes with smaller changes in the base prior model (e.g., expected quality improvement and completeness) showed increased posterior mass in the ROPE under the horseshoe prior, consistent with stronger regularization of weak signals. Estimates of the key secondary changes were stable across assumptions about the subject-level correlation structure (rank-1 factor, LKJ full covariance, rank-2 factor, rank-3 factor, and MGP shrinkage factor model).

### MNAR tipping-point sensitivity for the primary outcome.

Under MAR-based joint imputation, the posterior probability of a reduction in documentation time was Pr(Δ<0)=1.000. In MNAR pattern-mixture sensitivity analyses targeting the missing post values (8/52, 15.4%), no tipping point was observed up to δ=45 additional minutes added to missing post values. This held both for the MAR+δ adjustment and for the jump-to-baseline+δ scenario. Therefore, within the range explored, the posterior probability remained ≥0.95 even under extreme MNAR departures.

### Sampling diagnostics.

Across fitted Bayesian models, there were no divergent transitions and the maximum tree depth was not reached. Convergence for primary parameters was adequate (key [
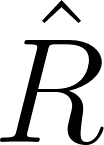
](https://www.codecogs.com/eqnedit.php?latex=%5Chat%7BR%7D#0) values approximately 1.00-1.01). In models with stronger shrinkage or higher-dimensional secondary structures, a small subset of secondary parameters showed weaker effective sample sizes, but this did not materially change the posterior summaries of the primary reduction or the large, consistent secondary effects reported above.

**References**

[1.](https://www.zotero.org/google-docs/?kJ3AXr)  [Cumming G. The new statistics: Why and how. Psychol Sci Sage Publications Sage CA: Los Angeles, CA; 2014;25(1):7–29.](https://www.zotero.org/google-docs/?kJ3AXr)

[2.](https://www.zotero.org/google-docs/?kJ3AXr)  [Ma Z, Chen G. Bayesian methods for dealing with missing data problems. J Korean Stat Soc Springer; 2018;47(3):297–313.](https://www.zotero.org/google-docs/?kJ3AXr)

[3.](https://www.zotero.org/google-docs/?kJ3AXr)  [Bolstad WM, Curran JM. Introduction to Bayesian statistics. John Wiley & Sons; 2016. ISBN:1-118-59315-4](https://www.zotero.org/google-docs/?kJ3AXr)

[4.](https://www.zotero.org/google-docs/?kJ3AXr)  [Kumar R, Carroll C, Hartikainen A, Martin O. ArviZ a unified library for exploratory analysis of Bayesian models in Python. J Open Source Softw Open Journals; 2019;4(33):1143.](https://www.zotero.org/google-docs/?kJ3AXr)

[5.](https://www.zotero.org/google-docs/?kJ3AXr)  [Carvalho CM, Polson NG, Scott JG. The horseshoe estimator for sparse signals. Biometrika JSTOR; 2010;465–480.](https://www.zotero.org/google-docs/?kJ3AXr)

[6.](https://www.zotero.org/google-docs/?kJ3AXr)  [Gelman A. Scaling regression inputs by dividing by two standard deviations. Stat Med Wiley Online Library; 2008;27(15):2865–2873.](https://www.zotero.org/google-docs/?kJ3AXr)

[7.](https://www.zotero.org/google-docs/?kJ3AXr)  [Lewandowski D, Kurowicka D, Joe H. Generating random correlation matrices based on vines and extended onion method. J Multivar Anal Elsevier; 2009;100(9):1989–2001.](https://www.zotero.org/google-docs/?kJ3AXr)

[8.](https://www.zotero.org/google-docs/?kJ3AXr)  [Bhattacharya A, Dunson DB. Sparse Bayesian infinite factor models. Biometrika Oxford University Press; 2011;98(2):291–306.](https://www.zotero.org/google-docs/?kJ3AXr)

[9.](https://www.zotero.org/google-docs/?kJ3AXr)  [Gelman A. Prior distributions for variance parameters in hierarchical models (comment on article by Browne and Draper). 2006;](https://www.zotero.org/google-docs/?kJ3AXr)

[10.](https://www.zotero.org/google-docs/?kJ3AXr)  [Abril-Pla O, Andreani V, Carroll C, Dong L, Fonnesbeck CJ, Kochurov M, Kumar R, Lao J, Luhmann CC, Martin OA. PyMC: a modern, and comprehensive probabilistic programming framework in Python. PeerJ Comput Sci PeerJ Inc.; 2023;9:e1516.](https://www.zotero.org/google-docs/?kJ3AXr)
