# Supplementary material for: Time Savings Through an AI Speech Assistant for Nursing Documentation: Pre-Post Time-Motion Study in German Long-Term Care
Source: J Med Internet Res. 2026 Apr 8;28:e86078. doi: 10.2196/86078 (PMC13061367; doi:10.2196/86078)
Supplement: Multimedia Appendix 5 [file jmir-v28-e86078-s005.docx]

# Multimedia Appendix 5

# Results of Exploratory Sensitivity Analyses

Across all model-specification robustness checks (Table S1), the adjusted change between baseline and post-implementation remained negative and of similar magnitude (approximately 14 to 15 minutes). Post-implementation documentation time was missing for 8/52 (15.4%) participants. MNAR departures targeting these cases indicated that conclusions were stable even under conservative departures from MAR.

### **Table S1.** Sensitivity analyses for the adjusted mean change in total documentation time (t_1_-t_0_), minutes

| **Model specification** | **β (min)** | **SE** | **df** | ***t*** | ***P* value** | **95% CI** |
| --- | --- | --- | --- | --- | --- | --- |
| Primary LMM (random intercepts: participant, facility; covariates: age, gender, baseline documentation type; N=52) | -15.00 | 3.36 | 46.29 | -4.46 | <.001 | -21.75 to -8.23 |
| Primary LMM (Complete-case only with both time points observed; n=44) | -14.51 | 3.33 | 43.00 | -4.35 | <.001 | -21.23 to -7.79 |
| Primary LMM + observer random intercept (N=52) | -14.99 | 3.37 | 46.29 | -4.45 | <.001 | -21.76 to -8.21 |
| Multiple imputation under MAR (m=40), pooled (N=52) | -14.81 | 3.25 | 92.85 | -4.56 | <.001 | -21.26 to -8.35 |
| Primary LMM + Cluster-robust SE (CR2), clustered by facility (N=52) | -15.00 | 4.00 | 10.50 | -3.75 | .003 | -23.86 to -6.14 |

## Effect modification by baseline characteristics

None of the time-by-baseline characteristic interaction terms were statistically significant (all *P*≥.24), indicating no reliable heterogeneity of the pre-post change by baseline age (Time×age: *β*=−0.35, SE 0.29; *t*_42.10_=−1.19; *P*=.24; 95% CI −0.93 to 0.24), gender (Time×gender: *β*=−0.80, SE 4.97; *t*_46.90_=−0.16; *P*=.87; 95% CI −10.80 to 9.21), or documentation type (contrast 1: *β*=5.21, SE 7.59; *t*_42.70_=0.69; *P*=.50; 95% CI −10.10 to 20.50; contrast 2: *β*=0.50, SE 5.13; *t*_42.80_=0.10; *P*=.92; 95% CI −9.84 to 10.80).

**MNAR sensitivity analyses**

MNAR analyses targeted participants with missing post-implementation documentation time (8/52, 15.4%) by modifying their imputed post values in the MAR-imputed datasets. Under jump-to-baseline (post := baseline) for these participants, the pooled adjusted mean change remained negative and statistically significant (β=-12.30 minutes, SE 2.91, *t*_98.00_=-4.22, *P*<.001; 95% CI -18.00 to -6.50). Across MAR δ adjustment scenarios (post := post + δ) evaluated over δ=0-45 minutes, no tipping point was observed. In contrast, in the Jump-to-baseline + δ (post := baseline + δ) scenario evaluated over δ=0-45 minutes, the tipping point was reached at δ=33 minutes. Thus, the pooled time effect remained statistically significant for δ=0-32 minutes.

### **ANCOVA-of-change**

In the pooled ANCOVA-of-change analysis, the mean change in total documentation time remained negative and statistically significant. The pooled mean change was -14.81 minutes (SE 3.28), *t*_29.95_=-4.51, *P*<.001, 95% CI -21.40 to -8.20.

### **MNAR tipping-point results for ANCOVA**

Under a jump-to-baseline assumption for participants missing post in the original dataset (post := baseline; δ=0), the adjusted mean change remained negative and statistically significant (-12.27 minutes, SE 2.36), t_46.12_=-5.20, *P*<.001, 95% CI -17.03 to -7.52. Tipping-point analyses targeted the 8/52 (15.4%) participants with missing post documentation time in the original dataset. Under MAR + δ adjustment (post := post + δ) departures, no tipping point was observed within the specified δ=0–45-minute grid. The first loss of statistical significance occurred at δ=52 minutes (*P*=.051), with the effect remaining statistically significant at δ=51 minutes (*P*=.044). Under Jump-to-baseline + δ (post := baseline + δ) departures, the first loss of statistical significance occurred at δ=36 minutes (*P*=.054), with statistical significance retained at δ=35 minutes (*P*=.047).
